# Risk of liver decompensation assessed in HIV/HCV co-infected individuals with advanced liver fibrosis: a faster countdown experience

**DOI:** 10.7448/IAS.17.4.19641

**Published:** 2014-11-02

**Authors:** Joao Pedro Alves, Susana Peres, Fernando Borges, Ana Cláudia Miranda, Teresa Baptista, Fernando Ventura, Isabel Antunes, Jaime Nina, Maria José Campos, Isabel Aldir, Kamal Mansinho

**Affiliations:** Serviço de Doenças Infecciosas e Medicina Tropical, Centro Hospitalar Lisboa Ocidental – Hospital de Egas Moniz, Lisboa, Portugal

## Abstract

**Introduction:**

Cirrhosis secondary to HCV infection is expected to peak in the next decade, particularly in the HIV co-infected subgroup and has become a leading cause of morbidity among these individuals. Efforts must be done to estimate the risk of liver decompensation (LD) in the short term, in order to define the appropriate time for HCV treatment.

**Materials and Methods:**

Retrospective observational cohort study aimed to assess the risk of LD among a group of HIV/HCV co-infected patients diagnosed in the past 23 years in a central hospital of Lisbon. Inclusion criteria: (1) advanced liver fibrosis ≥F3; (2) HCV treatment naïve or without sustained virologic response (SVR). Patients had a one to five years period of follow-up. Multiple linear regression, Mann-Whitney and Kendall were the statistical tests performed.

**Results:**

From 444 HIV/HCV co-infections, 66 met the inclusion criteria, with preponderance of male gender (82%), 35–45 years of age (55%), genotype 1a (52%), a mean of 13 years of co-infection and an AIDS stage documented in 65%, though the majority is under antiretroviral therapy (86%) and have TCD4+>500 µ/L (59%). Half (52%) showed evidence of steatosis, many of these (41%) presenting a history of alcoholism or overweight (BMI ≥25 Kg/m^2^). Pre-cirrhotic (F3 or F3/4) or cirrhotic (F4) stage was documented in 36 and 30 patients respectively. After staging, 28 (42%) initiated HCV treatment and SVR was achieved in 8 (29%) of those. Five (14%) pre-cirrhotic and twelve (40%) cirrhotic patients experienced at least one LD episode: 8 vs 28 cumulative events at five years and 2.8 vs 1.8 average years up to the first LD episode for pre-cirrhotic vs cirrhotic. The probability of remaining free of LD for pre-cirrhotic vs cirrhotic patients was 97% vs 78% (p≤0.01) at one year; 88% vs 65% (p≤0.001) at three years and 71% vs 44% (p≤0.001) at five years. Positive correlation was found between LD and the cirrhotic stage (vs pre-cirrhosis, p≤0.001), baseline AST ≥100 µ/L (vs <100 µ/L, p≤0.01) and platelet count <120 x 109/L (vs >120 x 109/L, p≤0.05).

**Conclusions:**

Cirrhosis accounts for a significant superior risk of LD. The time up to the first LD event differed in only one year between pre-cirrhotic and cirrhotic, standing for the importance of a rapid treatment referral in both subgroups. Modifiable risk factors that accelerate fibrosis are prevalent in HIV/HCV co-infected patients. Low platelet count, elevated AST and F4 stage predict the rapid progression to LD and the need for early HCV treatment. Large studies are required for further support of these results.

